# High-quality RNA extraction and the regulation of genes encoding cellulosomes are correlated with growth stage in anaerobic fungi

**DOI:** 10.3389/ffunb.2023.1171100

**Published:** 2023-07-17

**Authors:** Jennifer L. Brown, Taylor Gierke, Lazarina V. Butkovich, Candice L. Swift, Vasanth Singan, Christopher Daum, Kerrie Barry, Igor V. Grigoriev, Michelle A. O’Malley

**Affiliations:** ^1^ Department of Chemical Engineering, University of California, Santa Barbara, Santa Barbara, CA, United States; ^2^ US Department of Energy Joint Genome Institute, Lawrence Berkeley National Laboratory, Berkeley, CA, United States; ^3^ Department of Plant and Microbial Biology, University of California Berkeley, Berkeley, CA, United States; ^4^ Joint BioEnergy Institute, Lawrence Berkeley National Laboratory, Berkeley, CA, United States

**Keywords:** RNA, anaerobic, fungi, methanogens, CAZymes

## Abstract

Anaerobic fungi produce biomass-degrading enzymes and natural products that are important to harness for several biotechnology applications. Although progress has been made in the development of methods for extracting nucleic acids for genomic and transcriptomic sequencing of these fungi, most studies are limited in that they do not sample multiple fungal growth phases in batch culture. In this study, we establish a method to harvest RNA from fungal monocultures and fungal–methanogen co-cultures, and also determine an optimal time frame for high-quality RNA extraction from anaerobic fungi. Based on RNA quality and quantity targets, the optimal time frame in which to harvest anaerobic fungal monocultures and fungal-methanogen co-cultures for RNA extraction was 2-5 days of growth post-inoculation. When grown on cellulose, the fungal strain *Anaeromyces robustus* cocultivated with the methanogen *Methanobacterium bryantii* upregulated genes encoding fungal carbohydrate-active enzymes and other cellulosome components relative to fungal monocultures during this time frame, but expression patterns changed at 24-hour intervals throughout the fungal growth phase. These results demonstrate the importance of establishing methods to extract high-quality RNA from anaerobic fungi at multiple time points during batch cultivation.

## Introduction

Comparative transcriptome profiling is difficult to apply to non-model organisms as traditional nucleic acid extraction protocols and approaches do not often translate well to these systems (Shomron, 2021). This is particularly true when working with fungi that have extensive rhizoid or mycelial networks and chitin-rich cell walls (Orpin, 1977), which require difficult lysis and extraction protocols to isolate sufficient quantities of high-quality nucleic acids (Maaroufi et al., 2004; Fredricks et al., 2005; Edwards et al., 2017). For example, anaerobic fungi are non-model organisms that serve as a valuable source of diverse carbohydrate-active enzymes (CAZymes), with powerful biomass-degrading capabilities (Youssef et al., 2013; Edwards et al., 2017; Haitjema et al., 2017; Henske et al., 2017; Wilken et al., 2021). This means that they are particularly useful in biotechnology applications to generate value-added products from low-cost waste materials (Sanderson, 2011; Solomon et al., 2016b; Haitjema et al., 2017). Moreover, these fungi also produce unique natural products (Swift et al., 2021b; Swift et al., 2021a), which probably enables their function and which could be harnessed as an emerging class of antimicrobials or as therapeutic compounds. Several research teams have worked to develop unique lysis and extraction approaches to overcome challenges associated with obtaining high-quality genomic DNA from anaerobic fungi to access this biotechnology potential (Youssef et al., 2013; Calkins et al., 2016; Solomon et al., 2016b; Solomon et al., 2016c).

Although advancements to overcome the challenges associated with extracting DNA and RNA from non-model microbes, such as anaerobic fungi, have been made, universally effective RNA extraction methods are not yet well established for anaerobic fungi. For example, it is extremely challenging to extract similar quantities of high-quality RNA in the lag, exponential growth, and stationary phases that may prove relevant to deciphering the function of certain fungal genes (Solomon et al., 2014). Most gut fungal RNA studies to date have collected RNA data from one time point in the mid-log growth phase. These studies have focused on the differential regulation of CAZymes and/or the biosynthetic genes, which encode natural products within anaerobic fungi (Solomon et al., 2016a; Li et al., 2019; Swift et al., 2019; Brown et al., 2021), as altered by substrate, or cocultivation with other organisms. The ability to collect RNA sequencing (RNA-seq) data for a full-time course across all growth regimes provides valuable information regarding when the CAZyme and biosynthetic genes of interest are expressed. Determining how widely expression varies as a function of the growth phase would also inform bioreactor design to maximize the production of target products (e.g., enzymes or metabolites) produced by anaerobic fungi, either in isolation or in co-culture.

To monitor gene expression, a reliable method for the collection of high-quality RNA for transcriptomic analysis is needed. This study investigates how the time of harvest affects RNA quality, RNA concentration, and transcriptional regulation, with a focus on biomass-degrading enzymes and other fungal cellulosome components. We chose to examine fungal–methanogen co-cultures and fungal monocultures, as previous studies (Li et al., 2019; Swift et al., 2019; Brown et al., 2021) have demonstrated that the transcription of CAZymes increases at a given time point in the fungal growth phase when cocultivated with a methanogen. Fungal monocultures of *Anaeromyces robustus* and co-cultures of *A. robustus* and the methanogen *Methanobacterium bryantii* were cultivated on filter paper and harvested at 24-hour timepoints from the second day of growth to the seventh day of growth post inoculation for RNA extraction and subsequent RNA quality and quantity assessment. The optimal time frame in which to harvest anaerobic fungal monocultures and fungal-methanogen co-cultures for RNA extraction was 2-5 days of growth post-inoculation. During this window of growth, overall fungal CAZyme regulation in anaerobic fungal co-cultures with methanogens as compared with fungal monocultures was dependent on the time of harvest. The genes encoding fungal cellulosome components were upregulated in co-cultures of fungi and methanogens relative to fungal monocultures, with variation in expression occurring at 24-hour intervals. These findings highlight that timing and the phase of fungal growth are important factors to consider when designing experiments and deciphering transcriptomic regulation patterns.

## Methods

### Growing and harvesting cultures for RNA extraction

Anaerobic serum bottles (120 mL total volume) containing 80 mL of modified medium C (Theodorou et al., 2005) (“MC–”), with 0.8 mL of 100× vitamin solution (Teunissen et al., 1991) and 0.8 g of reed canary grass, were inoculated with cultures of the anaerobic fungus *A. robustus* (Solomon et al., 2016b; Haitjema et al., 2017) and the methanogen *M. bryantii*: 1.0 mL of *A. robustus* or a combination of 1.0 mL of *A. robustus* and 1.0 mL of *M. bryantii* [DSM No.-863, Deutsche Sammlung von Mikroorganismen und Zellkulturen (DSMZ)] (routine cultures were cultivated as described previously by Swift, et al.) (Swift et al., 2019). The reed canary grass was provided by the US Department of Agriculture, Agricultural Research Service, US Dairy Forage Research Center, and they were milled in a Model 4 Wiley Mill (Thomas Scientific) using a 4-mm screen size (courtesy of P. J. Weimer). The fungal and methanogen co-cultures and fungal monocultures were grown anaerobically at 39°C in Hungate tubes filled with 7.0 mL of autoclaved modified medium C (“MC–”) (Theodorou et al., 2005), containing 1.25 g/L yeast extract, 5 g/L Bacto™ Casitone, and 7.5 vol% clarified rumen fluid, with 0.08 g filter paper (Grade 3, 23 mm, 100 circles; CAT no. 1003–323, Lot No 16932763; Whatman GE Healthcare Life Sciences) as the growth substrate, supplemented with 0.1 mL of vitamin solution post autoclaving, and inoculated with 0.8 mL of the appropriate 80-mL inoculum culture at the mid-log growth phase (Teunissen et al., 1991). Pressure production was used as a proxy for fungal growth, as described previously (Theodorou et al., 1995). Daily pressure measurements were taken using a probe pressure transducer (Theodorou et al., 1995). Once methane was detectable in the co-cultures, indicating that a successful co-culture had formed (starting at 48 hours post-inoculation), three or four cultures were harvested at 24-hour intervals and stored for later RNA extraction. Endpoint methane measurements for co-cultures were taken from the headspaces of the culture tubes before harvesting the cultures. First, the pressure in each sample was measured using a pressure transducer (Theodorou, 1994), and the headspace composition was measured using a gas chromatograph (GC)-pulsed, discharge helium ionization detector (TRACE 1300; Thermo Fisher Scientific) (Cai and Stearns, 2013). Finally, the headspace pressure of the samples was vented to return the headspace to atmospheric pressure.

After sampling the headspace gas of the culture to determine if methane was present in the co-cultures, the cultures were opened in an anaerobic chamber and the colonized filter paper was transferred to a 15 mL Falcon™ tube containing 1 mL of RNAlater™ using sterilized tweezers. The Falcon™ tube was then removed from the anaerobic chamber and immediately stored at –80°C until later extraction. A volume of 5 mL of the culture supernatant was transferred to an Eppendorf tube and stored at –20°C for later high-performance liquid chromatography (HPLC) analysis.

### Extracting RNA from experimental samples

Samples were removed from storage at –80°C and thawed on ice. After thawing, the cell pellets of *A. robustus* fungal monocultures or *A. robustus* and *M. bryantii* co-cultures stored in RNAlater were spun down for 6 min at 4°C and 10,000 g and the RNAlater was removed. Cells were lysed by liquid nitrogen grinding. Total RNA was extracted using the RNeasy Mini Kit(QIAGEN) and a QIAcube by following the RNeasy Mini protocol for animal cells with QIAshredder homogenization and the optional on-column DNase digest. Samples were eluted in 50 µL of RNase-free water. An Agilent TapeStation (Agilent Technologies, Inc., Santa Clara, CA, USA) system was used to determine the quality of the sequenced RNA and a Qubit High Sensitivity RNA Assay was used to determine concentrations.

### RNA sequencing and data analysis

Stranded RNASeq library(s) were created and quantified by quantitative polymerase chain reaction (qPCR) analysis for both monoculture and co-culture samples. For differential gene expression analysis, sequencing of the libraries was carried out using the Illumina NovaSeq sequencer with NovaSeq XP V1 reagent kits and an S4 flow cell, following a 2 × 150 indexed run recipe. The filtered reads from each library were aligned to the *Anaeromyces robustus* genome using HISAT2, version 2.1.0 (Kim et al., 2015). Strand-specific coverage was generated using deepTools v3.1 (Ramírez et al., 2014). Raw gene counts were generated using featureCounts, with only primary hits assigned to the reverse strand included in the raw gene counts (Liao et al., 2014). Raw gene counts were used to evaluate the level of correlation between biological replicates using Pearson’s correlation and to determine which replicates would be used in the differential gene expression (DGE) analysis. Any replicate with a correlation above 0.85 qualified for inclusion in the analysis. At least three biological replicates for each condition were used for the RNA quality/quantity evaluation and for RNA-seq. DESeq2 (version 1.18.1) (Love et al., 2014) was subsequently used to determine which genes were differentially expressed between pairs of conditions. The parameters that were used to classify a gene as differentially expressed (DE) between conditions were a *p*-value < 0.05 and a log2-fold change greater than 1. Subsequent analysis was done using the filtered model gene catalog for *A. robustus* provided for download on the MycoCosm website (Grigoriev et al., 2014). Pre-ranked Gene Set Enrichment Analysis (GSEA) of regulated genes in co-cultures relative to fungal monocultures for each substrate condition was conducted using 1,000 permutations and weighted enrichment statistics (Mootha et al., 2003; Subramanian et al., 2005). The TOPCONS web server was used to determine the consensus prediction of membrane protein topology for upregulated and downregulated gene sets, In addition, sequences were annotated using Pfam and the HMMER web server (Finn et al., 2011; Tsirigos et al., 2015; Mistry et al., 2021). The Joint Genome Institute (JGI) MycoCosm portal was used to assign secondary metabolism cluster annotations, which were generated by the Secondary Metabolite Unknown Regions Finder (SMURF) algorithm (Khaldi et al., 2010; Grigoriev et al., 2014). The OrthoFinder tool, with default parameters, was used to generate ortholog predictions for *A. robustus* SM genes in three other anaerobic gut fungal strains (*Neocallimastix californiae*, *Caecomyces churrovis*, and *Piromyces finnis*) (Emms and Kelly, 2015; Haitjema et al., 2017; Mondo et al., 2017; Brown et al., 2021; Swift et al., 2021b). As there is no established cutoff value for biological relevance, this analysis was performed without instituting a transcript per million (TPM) cutoff value. The TPM values for differentially expressed genes are provided in the **Supplementary Data File** so that readers can assess whether or not this analysis would be biologically relevant for any future studies or applications.

### HPLC analysis

The levels of volatile fatty acids present in the supernatant of both co-cultures and monocultures were measured using an Agilent1260 Infinity HPLC (Agilent) system. The samples were prepared by acidifying to 5 mM, using sulfuric acid, and then incubating at room temperature for 5 min. Samples were then centrifuged for 5 min at 21,000 g. The supernatant was syringe filtered into an HPLC vial (Eppendorf FA-45–24–11) using a 0.22-µm polyvinylidene difluoride (PVDF) filter. The samples were analyzed using an Agilent 1260 Infinity HPLC (Agilent) system equipped with an autosampler unit (1260 ALS). The separation of formate, acetate, glucose, and lactate was carried out using a Bio-Rad Aminex^®^ 87H Ion Exclusion Column for organic acids (Part No. 1250140; Bio-Rad Laboratories, Inc., Hercules, CA, USA), with a mobile phase of 5 mM sulfuric acid. In-house standards were prepared with MC-blank culture medium as a base and sodium formate (ACS Grade, Fisher Chemical S648500), sodium acetate (ACS Grade, Fisher Chemical S210500), and L-lactic acid sodium (99%, extra pure; Acros Organics, 439220100), and D-(+)-glucose (Sigma-Aldrich Cat. No. G8270) at concentrations of 0.1 g/L and 1 g/L.

## Results and discussion

### High-quality RNA is extracted from anaerobic fungi and fungal–methanogen co-cultures 2–5 days post inoculation

High-quality RNA is the gold standard for transcriptomic studies as it remains unclear whether or not RNA degradation occurs uniformly across the transcriptome or at different rates (Gallego Romero et al., 2014). Degradation that is not uniform could result in inaccurate expression levels for genes of interest that do not accurately reflect *in vivo* production levels (Gallego Romero et al., 2014). Mechanical lysis through both bead beating and liquid nitrogen grinding has provided high-quality RNA for many previous transcriptional studies, effectively breaking through the chitin-rich rigid cell walls of anaerobic fungi to release nucleic acids (Haitjema et al., 2014). To prevent the heat generation associated with bead beating that leads to RNA degradation from occurring, in this study, liquid nitrogen was used to extract nucleic acids.

The fungal monocultures of *A. robustus* and fungal–methanogen co-cultures of *A. robustus* and the methanogen *M. bryantii* were grown on Whatman filter paper, a cellulosic substrate. RNA was extracted from fungal cultures harvested on days 2–7 post inoculation into batch anaerobic culture using a liquid nitrogen grinding lysis method. Although there is no universally accepted criterion to determine whether or not a given RNA sample is suitable for inclusion in a given study, quality metrics, such as RNA integrity number (RIN) (Schroeder et al., 2006), are often used to determine relative sample quality (Gallego Romero et al., 2014). Samples were sequenced on an Illumina NovaSeq sequencer from anaerobic fungal monocultures and fungal–methanogen co-cultures on days 2–5 post-inoculation into anaerobic batch cultures (co-culture samples from day 6 were also sequenced).

**Figure 1:**
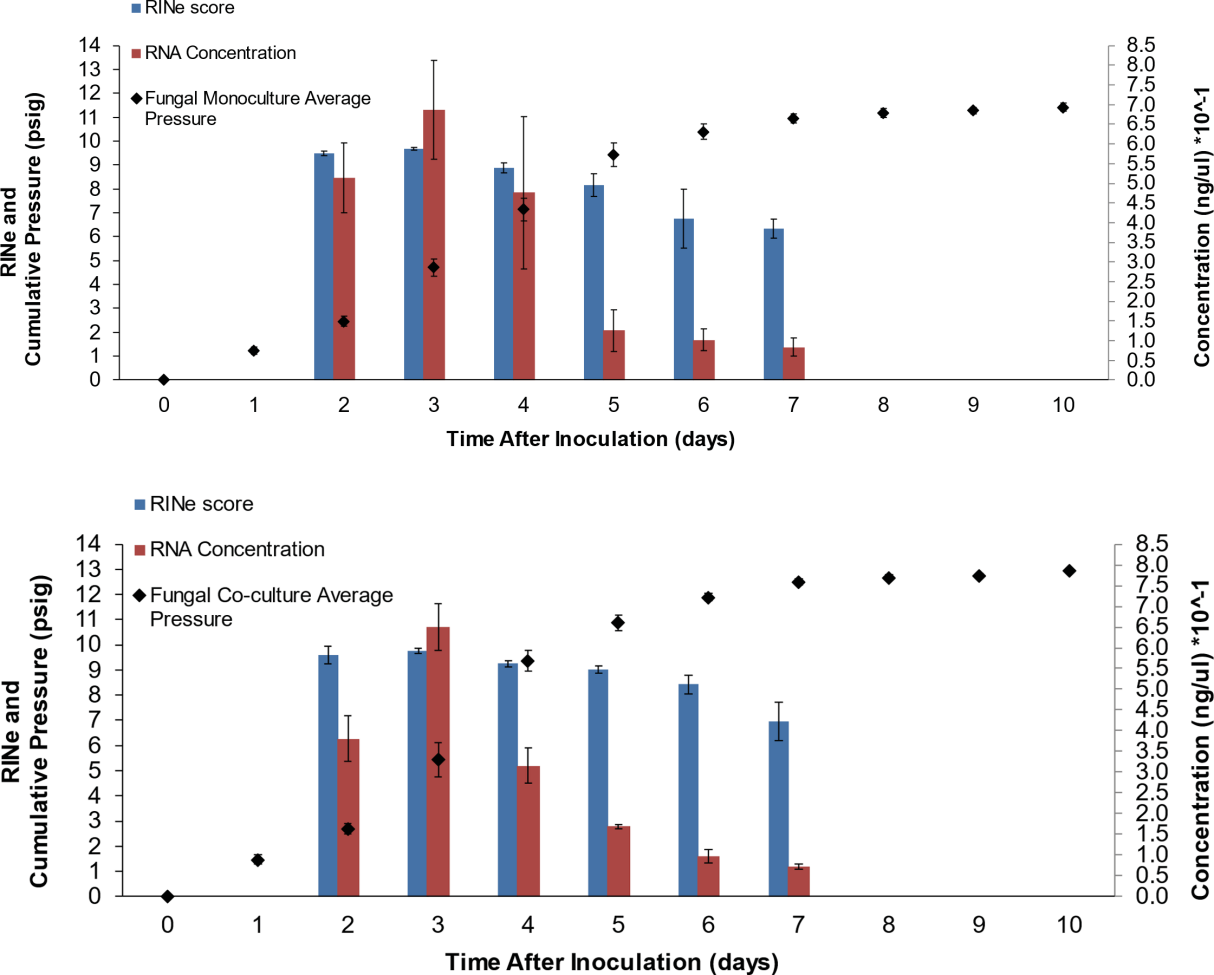
RNA concentrations and RINe scores for cultures harvested over 7 days of growth post-inoculation for fungal monocultures of *A. robustus* **(A)** and fungal-methanogen co-cultures of *A. robustus* and *M. bryantii* **(B)** both grown on a cellulose substrate (Whatman filter paper). RNA was extracted from cultures harvested on days 2-7 using a liquid nitrogen grinding lysis method. Samples were sequenced from both conditions on days 2-5 (co-culture samples from day 6 were also successfully sequenced). RNA degradation was more pronounced and RNA concentration decreased in cultures harvested on days 6 and 7, likely leading to the failure to sequence monoculture samples collected on day 6 and both monoculture and co-culture samples on day 7. The mean value is plotted for each set of replicates and error bars indicate standard deviation.

A plot of RNA concentrations and RNA integrity number equivalent (RINe) scores for cultures harvested over 10 days of growth is shown in **Figure 1A** (monocultures) and **Figure 1B** (co-cultures). RNA degradation was the most pronounced in the cultures harvested on days 6 and 7, which marks the beginning of the stationary phase based on accumulated pressure measurements, likely leading to the failure to sequence monoculture samples collected on day 6 and failure to sequence both the monoculture and co-culture samples on day 7. Although there is no generally accepted criterion for sample inclusion based on quality, samples with RINe scores as low as 3.95 have been included in previously published studies (Weis et al., 2007), and the average concentration of samples harvested on days 2–7 in this study exceeded that threshold. RIN and RINe values are both ranked on a scale from 1 to 10, with the highest value indicating no degradation; however, in contrast to RIN, RINe is a representation of the relative ratio of the signal in the fast zone to the 18S peak signal and provides a faster method of determining the total RNA integrity (Agilent Technologies, 2016). RIN and RINe have been shown to be equivalent for an Agilent 2200 TapeStation system and the Agilent R6K ScreenTape, when measuring RINe, and the Agilent 2100 Bioanalyzer, when measuring RIN (Agilent Technologies, 2016). The JGI recommends that samples submitted for sequencing have an RNA Quality Number (RQN) above 6.0 (Chovatia et al., 2018). All sample groups (days 2–7) had an average RINe score that exceeded this threshold (**Figure 1**).

RNA concentrations from both fungal monocultures and fungal–methanogen co-cultures were above 30 ng/µL in cultures harvested on days 2–4 during the exponential growth phase, then average concentrations decreased to half or less of that amount for days 5–7, marking the end of the exponential growth phase and the beginning of the stationary growth phase. The beginning of the stationary growth phase could have also contributed to the failure to sequence monoculture samples collected on day 6 and the failure to sequence both monoculture and co-culture samples on day 7. The JGI recommends that eukaryotic RNA samples in the low-input category have a concentration range of 10–1,000 ng/μL ( , ). The average concentration of fungal monoculture samples harvested on days 2–6 and the average concentration of fungal–methanogen co-cultures harvested on days 2–4 met this criterion (although the average concentration of co-cultures harvested on day 5 was extremely close—the average concentration for these samples was 9.7 ng/μL). These findings indicate that the optimal timepoint for RNA extraction from this anaerobic fungus ranges from day 2 to 5 post-inoculation, encompassing the exponential growth phase, based on both quantity and quality measures and whether or not the harvested samples could be sequenced. The fungus used in this study, *A. robustus*, is a polycentric fungus with multinucleate rhizomycelia (Haitjema et al., 2017; Mondo et al., 2017). This range could vary for other fungal strains or microbial pairings as a result of biological differences, such as the presence or absence of rhizoidal structures, as nuclei are present in the rhizomycelium of polycentric fungi (Tsai and Calza, 1992; Haitjema et al., 2014). Therefore, future studies should be conducted to determine whether this range for capturing sufficient RNA quantity/quality is generalizable across more anaerobic fungal genera.

### Overall CAZyme regulation in anaerobic fungal co-cultures depends on the time of harvest during the exponential growth phase

Multiple previous studies (Li et al., 2019; Swift et al., 2019; Brown et al., 2021) have found that fungal CAZymes are upregulated in cocultivation with a methanogen in multiple growth conditions, such as media formulation, substrate, or using a particular fungal strain. However, these studies used only one or at the most two time points of RNA collection during the growth phase of fungal monocultures and co-cultures, calling into question whether or not these findings would hold throughout the entire duration of co-culture cultivation (Li et al., 2019; Swift et al., 2019; Brown et al., 2021). One previous study investigated the transcriptional response in exponential gut fungal monocultures when pulsed with glucose for six time points over a relatively short 28-h time period (Solomon et al., 2016a). Recent research has determined that CAZymes are regulated at the mid-log growth phase and late growth phase of a gut fungal monoculture, and also in co-culture with a methanogen grown on glucose (Li et al., 2019), noting a change in CAZyme regulation between the growth phases. However, it remains unclear how differences in growth stage affect the outcome of CAZyme-focused transcriptional studies, and to what extent the timeline of cultivation drives the differences observed in these studies. Determining the optimal time frame for maximal expression of CAZymes is also crucial to informing bioprocessing strategies that seek to use anaerobic fungi, as the prevalence of CAZymes within a bioreactor determines the efficiency with which a batch culture can degrade plant biomass substrates.

DESeq2 was used to determine that 1,002 unique genes were differentially expressed (419 upregulated and 583 downregulated) by the anaerobic fungus *A. robustus* over the 4 days (days 2–5 post-inoculation) when examined in fungal–methanogen co-culture as compared with a fungal monoculture. Lists of upregulated and downregulated genes in the co-culture, as compared with a monoculture condition, can be found in the **Supplementary Data File**. Days 2–4 post inoculation fell within the exponential growth phase and day 5 post inoculation marked the beginning of the stationary growth phase based on measurements of pressure accumulation in the headspace of the cultures, which serves as a proxy for growth in the absence of quantitative methods to measure fungal cells grown on an insoluble substrate (Theodorou et al., 1995). Although the average accumulated pressure was slightly higher overall under co-cultivation, it did not appear to affect when the shift from exponential growth to stationary growth occurred relative to fungal monoculture. Out of the unique genes that were differentially expressed, 200 of those genes encoded fungal CAZymes. GSEA preranked analysis of CAZyme regulation revealed that CAZymes were enriched in upregulated genes in co-culture compared with monoculture on days 3 and 5 (significant at a false discovery rate, FDR, of < 25%), but not on days 2 and 4, as indicated in the **Supplementary Data File**. The day that cultures were harvested post-inoculation affected the total number of CAZymes regulated and whether or not more CAZyme genes were upregulated or downregulated when comparing co-cultures with monocultures. These findings reveal that the overall upregulation of fungal genes annotated as CAZymes in fungal–methanogen co-cultures relative to fungal monocultures observed in previous studies was likely to be highly dependent on the time of harvest.

These cultures were grown on filter paper, a cellulose substrate, and, therefore, results could vary if cultures are grown on other substrates, such as glucose or lignocellulose. Previous research has indicated that a common regulatory network for diverse CAZymes is upregulated for a variety of substrates; however, results from previous studies have also indicated that the gene expression levels of specific enzyme types for similar reactions were differentially regulated as a function of growth substrate (Solomon et al., 2016b). This indicates that a substrate-specific catabolic response also occurs in response to the presence of a particular growth substrate (Solomon et al., 2016b).

### Cellulosome components are transcriptionally upregulated in batch co-culture of fungi and methanogens with variation in expression at 24-hour intervals

The breakdown of biomass by anaerobic fungi is aided by extracellular fungal cellulosomes that consist of a catalytic complex that includes dockerins, carbohydrate-binding modules, and CAZymes grouped together for improved hydrolysis (Haitjema et al., 2017). A previous study examining one timepoint for RNA harvest indicated that growth on insoluble substrates such as filter paper, Avicel®, or reed canary grass induced expression of fungal cellulosomes for enhanced degradation in fungal monoculture (Solomon et al., 2016b). A second study found that co-culture of a non-rhizoidal fungal strain with a methanogen increased transcription of carbohydrate-binding modules and dockerin domains in co-cultures grown on cellulose (Avicel™) (Brown et al., 2021). We would therefore expect the expression of cellulosome components and the transcriptional upregulation of carbohydrate-binding modules and dockerins in co-culture to enhance the degradation capability of growth on the cellulosic filter paper substrate used in this study.

**Figure 2:**
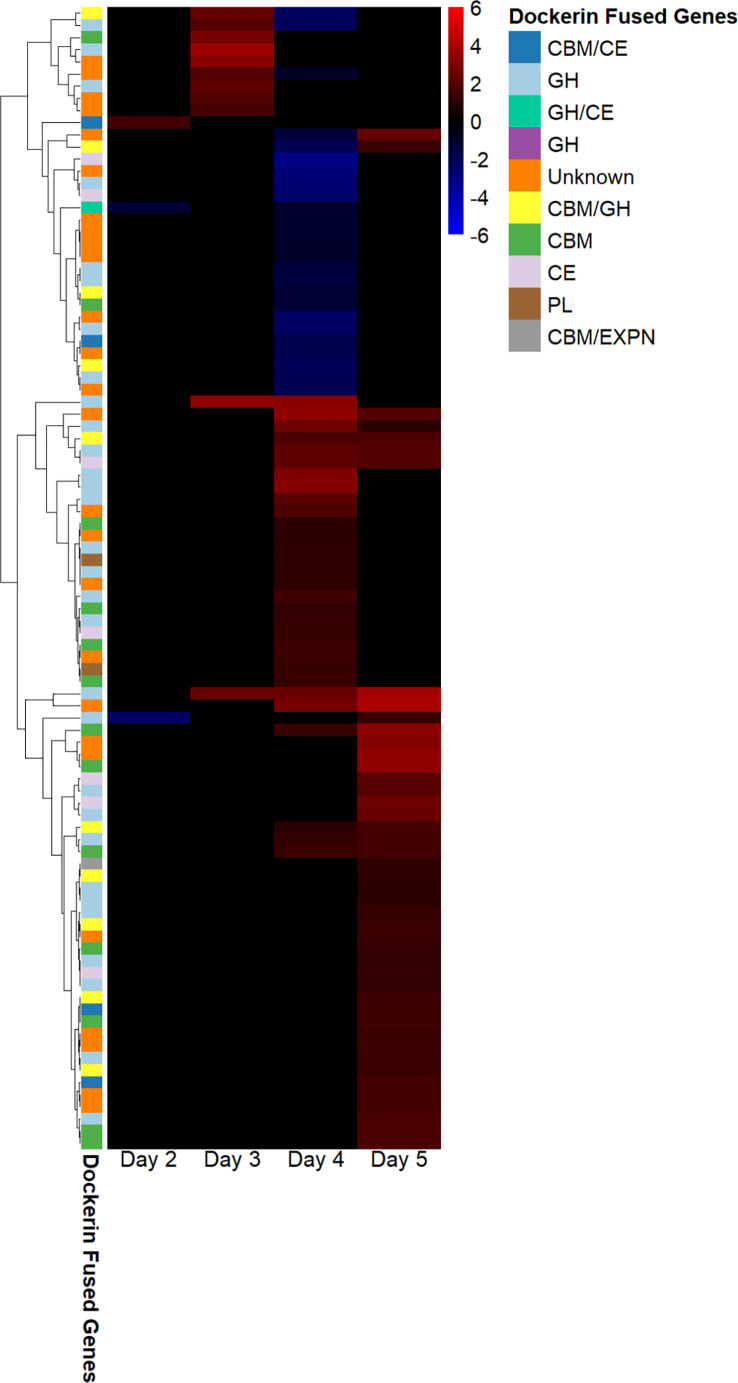
Dockerin regulation in *Anaeromyces robustus* and *Methanobacterium bryantii* co-culture as compared with *A. robustus* monoculture indicates that transcriptional upregulation of these cellulosome components is dependent on the time of batch culture harvest post inoculation. Regulated genes annotated as containing dockerin domains were upregulated on days 3 and 5—none were downregulated at these harvest timepoints, although the downregulation of genes annotated as containing dockerin domains was observed for cultures harvested on days 2 and 4. Regulation is determined using log2-fold change in expression, ranging from 6 to –6. The legend indicates which type of CAZyme the dockerin is fused to and if a CBM is also present. “Unknown” indicates that the dockerin is fused to a gene of unknown function. The transcriptional upregulation of these cellulosome components in fungal–methanogen co-cultures relative to fungal monocultures is dependent on the time of harvest for batch cultures grown on a cellulose substrate, with the regulation of largely unique dockerin-fused gene groups at harvest on a given day of growth post-inoculation. CAZyme, carbohydrate-active enzyme; CBM, carbohydrate-binding module.

We found that the regulation of fungal genes annotated as dockerins and carbohydrate-binding modules, and also the CAZymes glycoside hydrolases, carbohydrate esterases, glycosyl transferases, and polysaccharide lyases in fungal–methanogen co-culture relative to fungal monoculture varied at each 24-hour timepoint of the exponential growth phase. This indicates that cocultivation with a methanogen upregulates the expression of cellulosome components, which was the conclusion reached by previous studies, and is dependent on the time of harvest, as shown in **Figure 2**. These results suggest that co-culture with a methanogen bolsters the upregulation of cellulosome components observed previously in fungal monocultures grown on insoluble substrates, such as cellulose, at specific points in the growth phase. Only three genes annotated as CAZymes, carbohydrate-binding modules (CBMs), and/or as containing dockerin domains were upregulated under the cocultivation conditions on day 2. It is possible that this occurred because the fungus had not yet transcriptionally responded to the presence of the methanogen.

**Figure 3:**
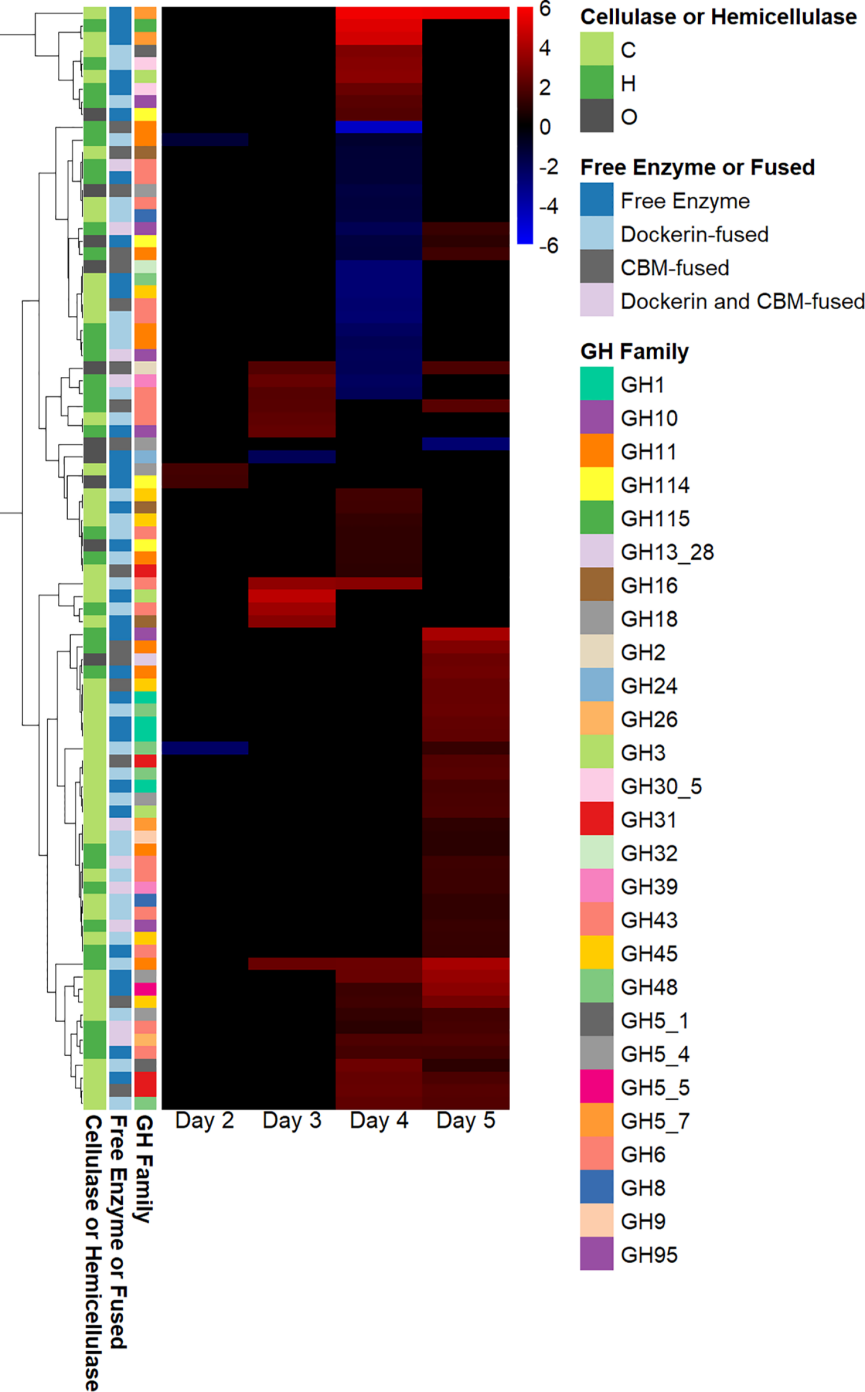
Glycosyl hydrolase regulation in co-cultures of *Anaeromyces robustus* and *Methanobacterium bryantii* as compared with *A. robustus* monoculture grown on a cellulose substrate reveals sequential upregulation of hemicellulase and cellulase enzymes. Although several genes annotated as GHs were downregulated on day 4 and two genes annotated as GHs were downregulated on day 2, only one gene annotated as a GH was downregulated on days 3 and 5—all other differentially expressed genes annotated as GHs were upregulated on days 3 and 5 in a regulation pattern similar to that observed for regulation of genes containing dockerin domains. The majority of genes annotated as GHs that were upregulated in fungal–methanogen co-culture on day 3 were hemicellulases, and the majority of genes annotated as GHs that were upregulated in fungal–methanogen co-culture on days 4 and 5 were cellulases. This observation could be attributed to an adaptive upregulation of enzymes in co-culture to free the core of plant biomass before cellulase regulation increases. Regulation is determined using log2-fold change in expression, ranging from 6 to –6. The legend indicates whether a given gene annotated as a GH is classified as a cellulase (C), a hemicellulase (H), or other (O) and whether the GH is a free enzyme or fused to a CBM and/or dockerin domain. GH, glycosyl hydrolase; CBM, carbohydrate-binding module.

Many fungal glycosyl hydrolases (GHs) assist in breaking down the cellulosic and hemicellulosic components of plant biomass (Murphy et al., 2011). Although several genes annotated as glycosyl hydrolases were downregulated on day 4, and two genes annotated as GHs were downregulated on day 2, most differentially expressed genes annotated as GHs were upregulated on days 3 and 5, as shown in **Figure 3**. The majority of genes annotated as GHs that were upregulated in fungal–methanogen co-culture on day 3 were hemicellulases, and the majority of genes annotated as GHs that were upregulated in the fungal–methanogen co-culture on days 4 and 5 were cellulases. As hemicellulases remove the hemicellulose in plant biomass to provide access to cellulose (Himmel et al., 2007), this observed pattern of regulation could be due to an adaptive upregulation of enzymes in co-culture to free the core of plant biomass before cellulase regulation increases, even though cellulose was the only substrate present in this experiment (Solomon et al., 2016b).

The removal of hemicellulose from plant biomass to free cellulose is accompanied by pectin removal by polysaccharide lyases (PLs) and carbohydrate esterases (CEs). The regulation of genes annotated as CEs and PLs is shown in **Supplemental Figures 1** and **2**. Although 11 genes annotated as CEs were downregulated on day 4 of growth post-inoculation, genes annotated as CEs and PLs were only upregulated in the cocultivation condition on days 3 and 5 of growth post-inoculation. Specifically, six genes annotated as CEs and one gene annotated as PL were upregulated on day 3 of growth. In addition, 11 genes annotated as CEs and one gene annotated as PL were upregulated on day 5 of growth. This demonstrates that cocultivation with a methanogen increases the transcription of genes associated with the pectin-removal process of biomass breakdown in the presence of cellulose, even if pectin is absent. GTs were only downregulated (exclusively on day 4) and not regulated on any of the other days, as shown in **Supplemental Figure 2**. These findings collectively indicate that CAZyme production may not be consistent over the entire exponential growth phase. The transcriptional regulation on days 3 and 5 would suggest that the previously observed patterns of cellulase and cellulosome component upregulation on insoluble substrates, such as filter paper, are enhanced by cocultivation with a methanogen, as indicated by previous studies that examined one or two specific timepoints only.

### Fungal secondary metabolite core genes are upregulated in early growth with slight elevations of primary metabolites glucose and acetate in co-culture relative to monoculture in late growth

In addition to CAZyme regulation, the regulation of biosynthetic genes has also been examined in transcriptional studies (Swift et al., 2021b; Swift et al., 2021a). For this analysis, we considered the predicted core and accessory SM gene annotations, where core genes encode SM biosynthetic enzymes and accessory genes encode supporting functions, such as tailoring enzymes, transport, and self-resistance. There were 45 putative biosynthetic gene clusters with 14 total polyketide synthase (PKS) or PKS-like core genes in the *A. robustus* genome. In total, there were 47 predicted core genes and 70 predicted accessory genes across all biosynthetic gene clusters, as shown in the **Supplementary Data File**. It is traditionally thought that secondary metabolite production occurs during the stationary growth phase of a microbe (Ruiz et al., 2010). However, in agreement with previous work (Swift et al., 2021b; Swift et al., 2021a), in both the *A. robustus* monoculture and in the *A. robustus* and *M. bryantii* co-culture, we observed the upregulation of only a few putative biosynthetic genes in *A. robustus* at later days in the growth phase. Contrary to expectations, all 14 PKS/PKS-like core genes in the *A. robustus* genome were upregulated in early *A. robustus* monoculture, and 13 out of the 14 genes were upregulated in co-culture with the methanogen *M. bryantii*. In addition, of the 10 most highly expressed predicted SM core genes, a majority were significantly upregulated at earlier growth (day 2) compared with late growth under both monoculture (day 5) and co-culture conditions (day 6), as shown in the **Supplementary Data File**. These findings, in agreement with previous studies, suggest that anaerobic fungi may be unique in their tendency to transcriptionally upregulate biosynthetic genes early in the growth phase before other microbes typically make the metabolic shift to secondary metabolite production in the stationary growth phase.

In the synergistic relationship that exists between fungi and methanogens, the methanogens remove hydrogen produced by the fungi and convert it to methane, resulting in the increased fungal production of acetate, formate, lactate, and ethanol over time. No significant differences were observed in ethanol, lactate, and formate levels between the monocultures of *A. robustus* and co-cultures of *A. robustus* and *M. bryantii* during this experiment, as shown in **Supplemental Figures 3–5**. In co-cultures, we observed slightly higher acetate and glucose levels than in monocultures on day 5, as shown in **Supplemental Figure 6**. A lack of statistically significant differences among the fermentation products is largely in agreement with what has been observed previously in a comparison of fermentation products produced in the monocultures of *A. robustus* with the co-cultures of *A. robustus* and *M. bryantii*, except that lactate was detected in the co-cultures but not in the monocultures on the third day of growth on a reed canary grass substrate. Although there were higher levels of acetate present in co-cultures on day 5 of this experiment, there was no statistically significant difference in acetate levels on day 3 (Swift et al., 2019).

Notably, the methanogen *M. bryantii* does not appear to utilize formate in this fungal–methanogen pairing grown for 5 days on filter paper. This contrasts with previous studies, in which *M. bryantii* was observed to utilize the formate produced by the fungus in a pairing of *M. bryantii* with *A. robustus* grown on filter paper for 10 days (Gilmore et al., 2019), and another study in which *M. bryantii* was observed to utilize formate produced by the fungus in a pairing of *M. bryantii* with *C. churrovis* grown on Avicel, reed canary grass, glucose, fructose, and xylan (Brown et al., 2021). This observation implies that the previous hypothesis that co-culture with rumen anaerobic fungi stimulates formate utilization by inducing the function of a formate transporter and formate dehydrogenase in the *M. bryantii* genome (Ruiz et al., 2010) may not be applicable until much later in the growth phase for certain strains or growth conditions.

## Conclusions

In this study, we have demonstrated the importance of designing transcriptional studies of anaerobic fungi that sample the entirety of the lag, exponential, and stationary growth phases. In addition, we have established a method and timeframe for the extraction of high-quality RNA from the anaerobic fungal strain *A. robustus* grown on a cellulose substrate. Although there is value in determining gene expression for given conditions at a fixed point in time, we have shown, for the anaerobic fungus *A. robustus*, that the expression and upregulation of genes of biotechnological interest under cocultivation conditions with the methanogen *M. bryantii*, such as CAZymes and biosynthetic genes, vary throughout the growth phase. These findings have implications for bioreactor design or future studies to identify secondary metabolites, as this study has shown that timing could be crucial in harnessing the potential of anaerobic fungi and perhaps of other anaerobic microorganisms in these types of applications.

## Data availability statement

The original contributions presented in the study are publicly available. This data can be found here: The National Center for Biotechnology Information (NCBI) BioProject PRJNA887131.

## Author contributions

JB and MO’M designed the study, analyzed the data, and edited the manuscript. JB carried out experiments and RNA extractions. JB, TG, LB, CS, VS, CD, KB, and IG sequenced or analyzed the transcriptomic sequencing data. TG and JB analyzed HPLC data. JB and MO’M wrote and revised the paper. All authors contributed to the article and approved the submitted version.
