# Supplementary material for: High-quality RNA extraction and the regulation of genes encoding cellulosomes are correlated with growth stage in anaerobic fungi
Source: Front Fungal Biol. 2023 Jul 17;4:1171100. doi: 10.3389/ffunb.2023.1171100 (PMC10512310; doi:10.3389/ffunb.2023.1171100)
Supplement: Supplementary file 2 [file Image_1.pdf]

Supplement

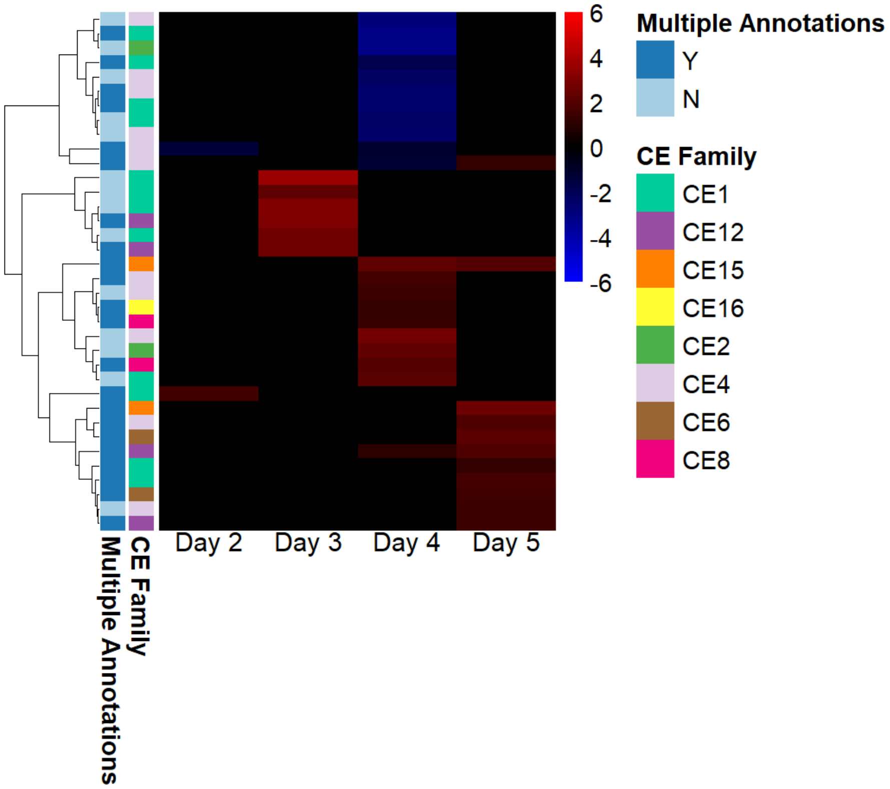

Supplemental Figure 1. Regulation of fungal genes annotated as carbohydrate esterases (CE) in *A. robustus* and *M. bryantii* fungal-methanogen co-culture versus *A. robustus* fungal monoculture. Regulated genes annotated as CEs were only upregulated (none downregulated) on days 3 and 5. Regulation is determined using log2fold change in expression, ranging from 6 to -6.

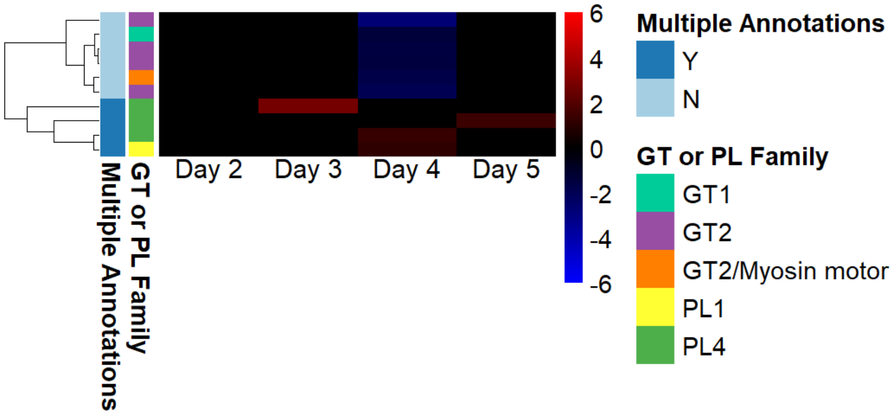

Supplemental Figure 2. Regulation of fungal genes annotated as glycosyl transferases (GT) and polysaccharide lyases (PL) in *A. robustus* and *M. bryantii* fungal-methanogen co-culture versus *A. robustus* fungal monoculture. Regulation is determined using log2fold change in expression, ranging from 6 to -6. GTs were only downregulated and PLs were only upregulated on any of the days cultures were harvested post-inoculation.

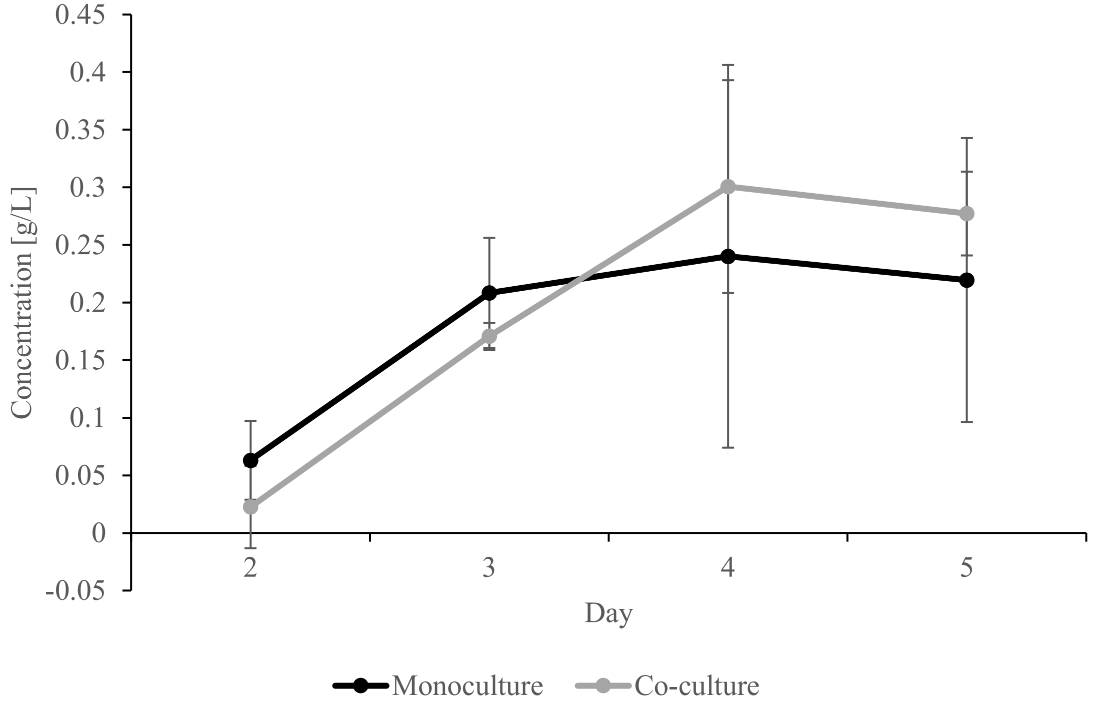

Supplemental Figure 3. Formate concentration over time in monocultures of *A. robustus* and co-cultures of *A. robustus* and *M. bryantii* grown on a cellulose substrate. The mean value is 19 plotted for each set of replicates and error bars indicate standard deviation.

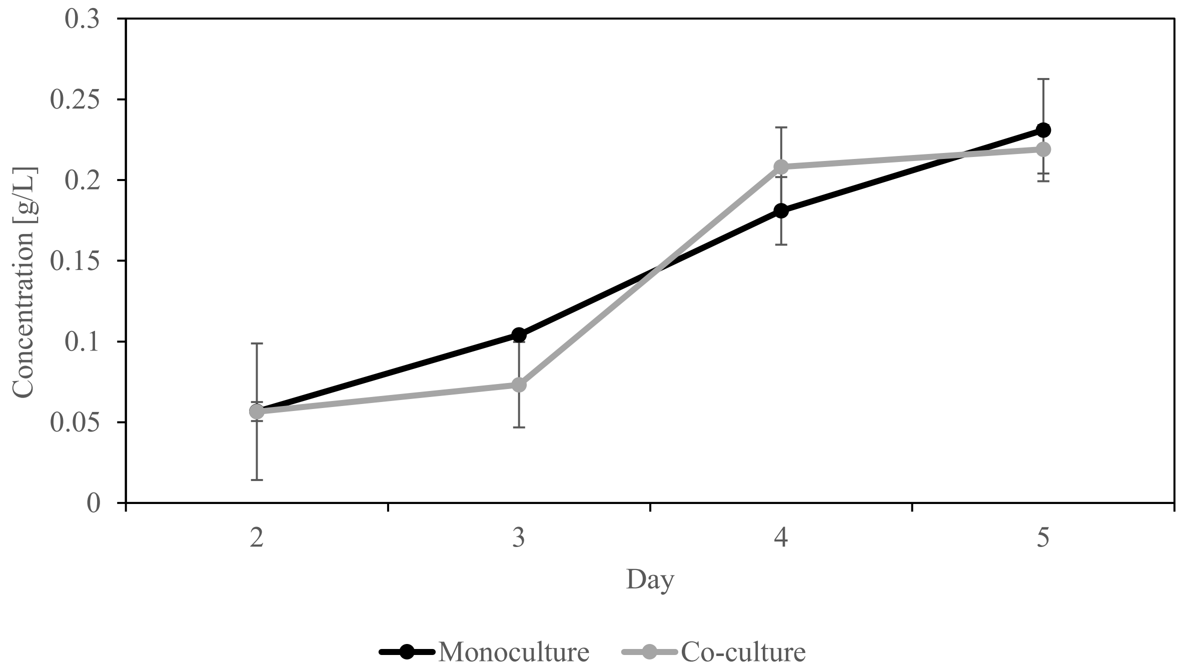

Supplemental Figure 4. Lactate concentration over time in *A. robustus* monocultures and *A. robustus* and *M. bryantii* co-cultures grown on a cellulose substrate. The mean value is plotted for each set of replicates and error bars indicate standard deviation.

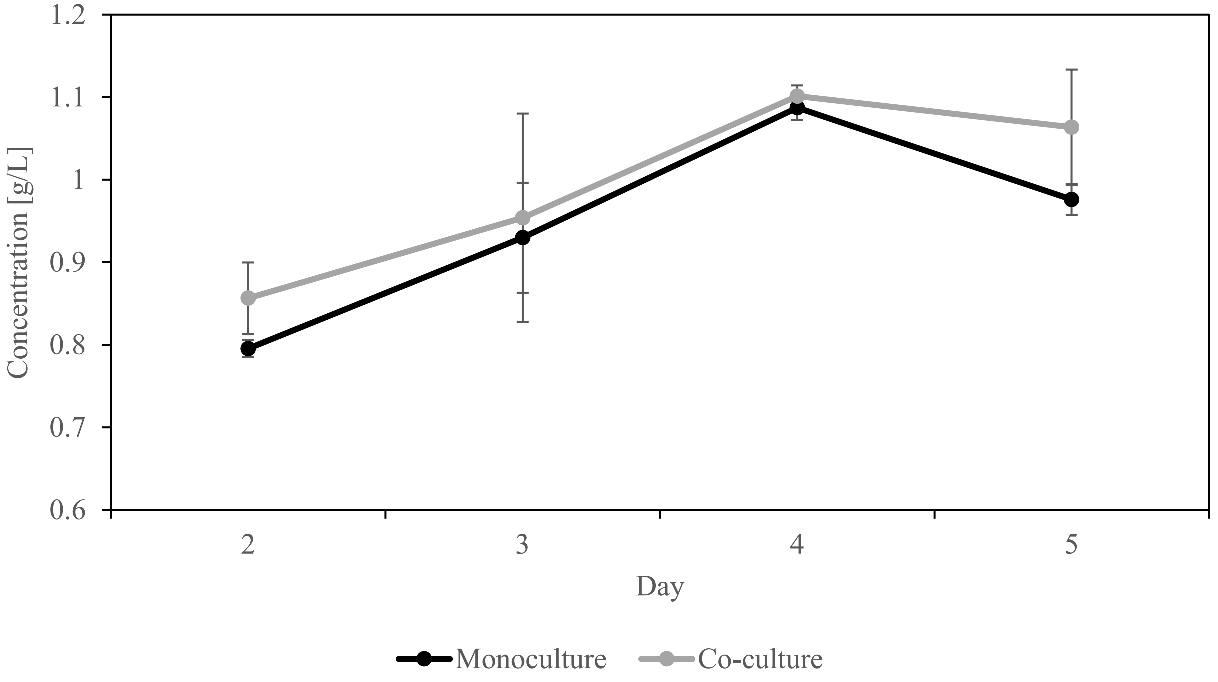

Supplemental Figure 5. Ethanol concentration over time in *A. robustus* monocultures and *A. robustus* and *M. bryantii* co-cultures grown on a cellulose substrate. The mean value is plotted for each set of replicates and error bars indicate standard deviation.

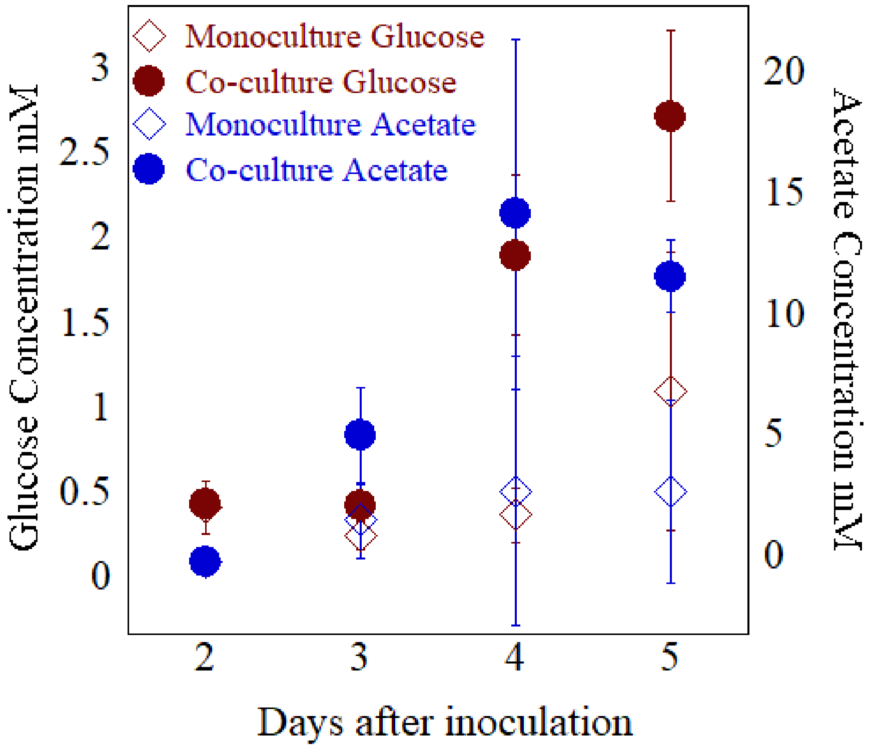

Supplemental Figure 6. Co-cultivation of *A. robustus* with a methanogen increased acetate production and glucose release when grown on a cellulose substrate. Higher levels of glucose and acetate were present in *A. robustus* and *M. bryantii* co-cultures compared to *A. robustus* monocultures after 5 days of growth on a cellulose substrate (Whatman filter paper). The mean value is plotted for each set of replicates and error bars indicate standard deviation.
